# Novel variants underlying autosomal recessive neurodevelopmental disorders with intellectual disability in Iranian consanguineous families

**DOI:** 10.1002/jcla.24241

**Published:** 2022-01-12

**Authors:** Mahdiyeh Moudi, Mohammad Yahya Vahidi Mehrjardi, Hossein Hozhabri, Zahra Metanat, Seyed Mehdi Kalantar, Mohsen Taheri, Nasrin Ghasemi, Mohammadreza Dehghani

**Affiliations:** ^1^ Department of Genetics Shahid Sadoughi University of Medical Sciences Yazd Iran; ^2^ Genetics of Non‐Communicable Disease Research Center Zahedan University of Medical Sciences Zahedan Iran; ^3^ Medical Genetics Research Center Shahid Sadoughi University of Medical Sciences Yazd Iran; ^4^ Medical Bioinformatician Centogene Rostock Germany; ^5^ Department of Genetics School of Medicine Zahedan University of Medical Sciences Zahedan Iran; ^6^ Abortion Research Centre Yazd Reproductive Sciences Institute Shahid Sadoughi University of Medical Sciences Yazd Iran

**Keywords:** *FBXO31*, neurodevelopmental disorders, *TIMM50* and *CEP290*

## Abstract

**Background:**

Intellectual disability (ID) is a heterogeneous group of neurodevelopmental disorders that is characterized by significant impairment in intellectual and adaptive functioning with onset during the developmental period. Whole‐exome sequencing (WES)‐based studies in the consanguineous families with individuals affected with ID have shown a high burden of relevant variants. So far, over 700 genes have been reported in syndromic and non‐syndromic ID. However, genetic causes in more than 50% of ID patients still remain unclear.

**Methods:**

Whole‐exome sequencing was applied for investigation of various variants of ID, then Sanger sequencing and *in silico* analysis in ten patients from five Iranian consanguineous families diagnosed with autosomal recessive neurodevelopmental disorders, intellectual disability, performed for confirming the causative mutation within the probands. The most patients presented moderate‐to‐severe intellectual disability, developmental delay, seizure, speech problem, high level of lactate, and onset before 10 years.

**Results:**

Filtering the data identified by WES, two novel homozygous missense variants in *FBXO31* and *TIMM50* genes and one previously reported mutation in the *CEP290* gene in the probands were found. Sanger sequencing confirmed the homozygote variant's presence of *TIMM50* and *FBXO31* genes in six patients and two affected siblings in their respective families. Our computational results predicted that the variants are located in the conserved regions across different species and have the impacts on the protein stability.

**Conclusion:**

Hence, we provide evidence for the pathogenicity of two novel variants in the patients which will expand our knowledge about potential mutation involved in the heterogeneous disease.

## 1 INTRODUCTION

Next‐generation sequencing (NGS) methods have revolutionized the neurodevelopmental disorder diagnosis, including intellectual disability (ID). These methods have accelerated the identification of causative genes and variants involved in the ethology of disease.^1^ ID is a heterogeneous neurodevelopmental disorder characterized by significant impairment in intellectual and adaptive functioning with onset during the developmental period. By a total prevalence of 1%–3%, the disease has extensive phenotypic variability and genetic heterogeneity worldwide.^2^

As of 2016, some papers showed that over 700 genes were reported in syndromic and non‐syndromic ID.^1^ Ghandil et al reported a novel homozygous variant in an Iranian family with syndromic ID.^3^ Therefore, NGS has increased the number of causative genes liked to ID facilitating in the diagnosis of the patients.^4^

The clinical features of syndromic ID include mild‐to‐severe intellectual disability, seizure, microcephaly, neuropsychiatric disorders, epilepsy, and motor dysfunction, and dysmorphic features.^5^ The clinical and molecular heterogeneity can pose a major challenge in the molecular diagnosis of ID. Many studies showed that the diagnostic yield of causative variants in ID patients with variable severity is low and changeable, ranging from 16% to 68% using whole‐exome sequencing (WES).^6^ Several factors such as the reduced penetrance, variable phenotype and syndromic nature of the disease, and lack of data in other family's members are the causes of the low‐yield of diagnostic tests. In contrast, WES‐based studies in consanguineous families with individuals affected with ID showed a high diagnosis rate of potential variants. For example, one paper posited that the WES approach's diagnostic yield in Middle East Asia was up to 90%.^7^

Using WES, we found two novel homozygous variants in *FBXO31* and *TIMM50* genes in four Iranian consanguineous families diagnosed with autosomal recessive neurodevelopmental disorders with intellectual disability. Besides, one known homozygous variant, which were previously reported, was identified in another family. These results were confirmed by Sanger sequencing in each family.

## 2 METHODS

### 2.1 Editorial policies and ethical considerations

The study was approved by the ethics committee of Shahid Sadoughi University of Medical Sciences department (IR.SSU.MEDICINE.REC.1399.199). Written informed consent forms for publishing and participating were obtained from all family members before the study. Five families with ten patients from Baluch region were recruited in Ali Asghar hospital, Zahedan, Iran.

### 2.2 Whole‐exome sequencing

According to the manufacturer's instructions, after collecting blood samples from all family members, DNA extraction was carried out by the QIAamp DNA Mini Kit. We performed library preparation and sequencing on probands of each family (V‐6) using the SureSelect Human All Exon V6 kit (Agilent Technologies) and HiSeq4000 machine sequencer from Illumina with the coverage and sensitivity of 100X and >99%, respectively. IlluQC.pl (SCR_005461) and Cutadapt software^8^ were applied for filtering the raw data. Then, Burrows Wheeler Aligner (BWA) tool was used to align reads to the reference human genome.^9^ We perform post‐alignment and variant calling using Picard (SCR_006525) (http://broadinstitute.github.io/picard/), the Genome analysis tool kit (GATK, RRID:SCR_001876),^10^ SAM tools (SCR_005227),^11^ HaplotypeCaller,^12^ and ANNOVAR (SCR_012821) software.^13^ In filtering strategy, we run a list of the related genes of ID on TSV files, which was provided the published data, gene panels, and DisGenet database. Then, the synonymous, intronic, and benign variants were removed from the filtered files, and the functional variants were prioritized according to their function and inheritance mode. The shortlisted variants' pathogenicity was checked using Varsome, InterVar, and ClinVar (SCR_006169) databases. Also, the candidate variants were compared with Iranome project, which is a catalog of genomic variations on 800 individuals from eight major Iranian ethnic groups. So, the project is useful to filter out the population‐specific variants.^14^

### 2.3 Sanger sequencing

Gene Runner software was used for designing the proper sequencing primers for confirming the variants in the five families (Table 1). DNA was amplified with PCR by designed primers for shortlisted variants, and Sanger sequencing was carried out by BigDye™ Terminator v3.1 Cycle Sequencing Kit and ABI‐3700 DNA analyzer (Thermo Fisher).

**TABLE 1:** Primer sequences of CEP290, FBXO31, and TIMMP50 genes

No	Primer name	Sequence	Product size (bp)
1	CEP290‐F	TGGCATATGAAGAACCAGG	440
2	CEP290‐R	TGTGATCTCGTTGTAAATTAGC	
3	FBXO31‐F	AGTGTTTCAGCATTGTGC	620
4	FBXO31‐R	CTGCTTCTATTCACAGGTCAG	
5	TIMMP50‐F	GTTCCTATCTGGCGGTATG	605
6	TIMMP50‐R	AGAGTGGGTGTTAGCATG	

### 2.4 Bioinformatics analysis

As the 3‐D structures of *TIMM50* and *CEP290* proteins have not been identified in the protein data bank (PDB, SCR_012820), Swiss‐Model online (SCR_013032) (https://swissmodel.expasy.org/) and I‐Tasser (SCR_014627) (https://zhanglab.dcmb.med.umich.edu/I‐TASSER/) were performed to find the homology modeling (UniProt ID: Q3ZCQ8 and O15078). These servers are the computational tools to predict the protein structures and functions based on the homologous structures and energy minimization. The best model was selected as highly acceptable scores based on the following parameters: PROCHECK's Ramachandran plot, ERRAT, verify 3D, and Clash score analyses. The crystal structure of FBXO31 protein was obtained from the PDB bank database (https://www.rcsb.org/) (PDB ID:5VZT). Phyre2 online tool (http://www.sbg.bio.ic.ac.uk/phyre2/html/page.cgi?id=index) and PyMOL software (2–5–0 version, SCR_000305) were used to check the possible effects of novel variants on the normal protein structure model. Consurf software (SCR_002320) (https://consurf.tau.ac.il/) was applied to align across orthologous sequences from different species to evaluate conserved regions in the protein structures. This server indicated the evolutionary conservation scores as color codes in each protein structure (ranging from blue to purple), where blue and purple colors showed the lowly and highly conserved positions, respectively. Several algorithms such as DynaMut,^15^ mCSM,^16^ SDM,^17^ and DUET^18^ were performed to predict the impact of variant on protein stability which are measuring the changes in free energy (ΔΔG) for each of the variants in the protein sequences.

## 3 RESULTS

### 3.1 Case presentation

#### 3.1.1 Pedigree I

**FIGURE 1:**
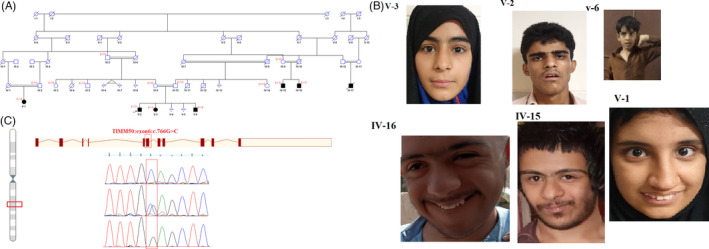
(A) Pedigree of three consanguineous families. Affected individuals are shown by dark symbols. (B) photographs of V‐1, 2, 3, 6, 15, and 16. (C) Electropherograms indicate the homozygous wild‐type sequence, heterozygous, and the homozygous c.766G>C in *TIMM50* gene

There was a history of six members affected with ID in three families born from healthy and consanguineous parents (V‐1, V‐2, V‐3, V‐6, IV‐15, and IV‐16 in Figure 1A,B). The proband (V‐2) is an 18‐year‐old male referred to the genetic counseling center with various clinical features, including seizure, severe intellectual disability, lack of speech, delayed psychomotor development, failure to thrive, delayed walking, absent speech, aggressive behavior, and increased activity serum lactate (Table 2). Therefore, other affected family members developed growth delay, recurrent episodes of seizures within the first month of life, failure to thrive, and increased serum lactate. Thus, they could not raise their head at 7–8 months of age and walk or sit when they are 3–4 years old. Laboratory results like karyotype and brain MRI tests were all normal.

**TABLE 2:** Clinical features of the described individuals

Pedigree	Pedigree 1		Pedigree 2		Pedigree 3					
Patient	VI−1	VI−3	V−1	V−2	V−2	V−3	V−6	V−1	V−15	V−16
Age at last exam	14 years	5 years	15 years	12 years	18 years	15 years	7 years	14 years	22 years	20 years
Gender	Female	Male	Female	Male	Male	Male	Male	Female	Male	Male
Intellectual disability	Moderate	Moderate	Severe	Severe	Severe	Moderate	Severe	Severe	Severe	Severe
Microcephaly	Mild	Mild	‐	‐	‐	‐	‐	‐	‐	‐
Seizure	‐	‐	+	+	+	‐	+	+	+	+
Developmental delay	‐	‐	+	+	+	+	+	+	+	+
Prominent supraorbital ridges	+	+	‐	‐	‐	‐	‐	‐	‐	‐
Long face	+	+	‐	‐	‐	‐	‐	‐	‐	‐
Prominent jaw	+	+	‐	‐	‐	‐	‐	‐	‐	‐
Prominent lips	+	+	‐	‐	‐	‐	‐	‐	‐	‐
Visual impairment	‐	‐	+	+	‐	‐	‐	‐	‐	‐
Speech	Delay	Delay	Sound	Sound	Sound	Delay	Delay	Sound	Sound	Sound
Kidney disease	‐	‐	‐	+	‐	‐	‐	‐	‐	‐
Brain MRI	Normal	Normal	Vermis aplasia	Normal	Normal	Normal	Normal	n.r	n.r	n.r
Karyotype	Normal	Normal	Normal	Normal	Normal	Normal	Normal	Normal	Normal	Normal
Metabolic test	‐	‐	‐	‐	⬆ lactate	⬆ lactate	⬆ lactate	⬆ lactate	⬆ lactate	⬆ lactate

Abbreviations: ‐, negative; +, positive; ⬆, high; n.r, not reported.

#### 3.1.2 Molecular analysis

**FIGURE 2:**
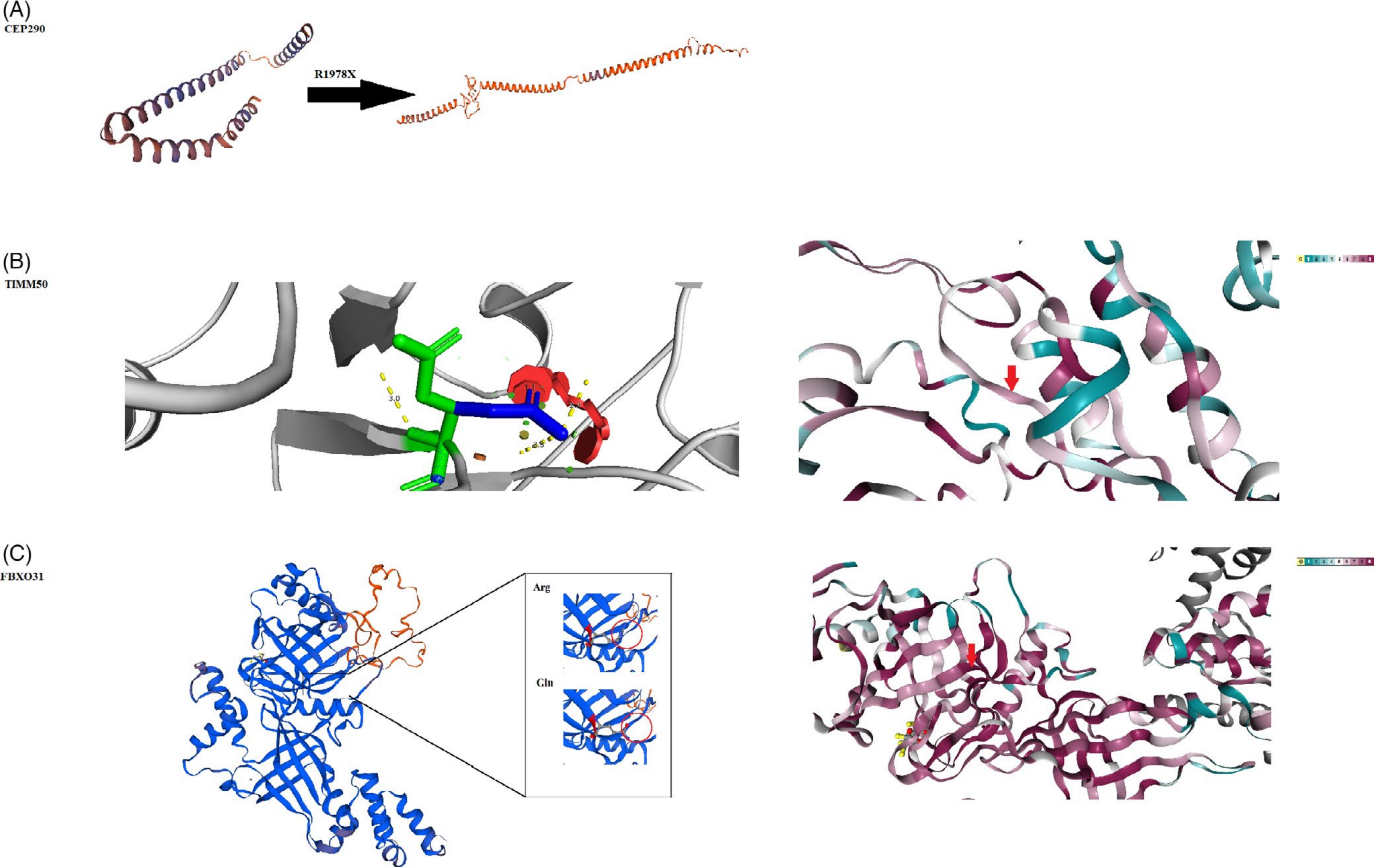
In silico analyses of the *TIMM50*, *FBXO31*, and *CEP290* genes. (A) Phyre2 investigator indicated that the p. R1978X variant causes premature protein by stop codon at position 1978 of *CEP290* sequence. (B) left: PyMol software predicted that the mutant acid amine creates a clash (red color) with other residues in the enlarged image. Right: the schematic structure of conservation in the different positions of TIMM50 protein using ConSurf server, in which glutamate residue is located in conserved region (score =7). The purple and green colors show a highly and lowly conserved region, respectively. (C) left: the mutant variant causes a change in the beta‐sheet structure of this region through Phyre 2 investigator. Right: the amino acid sequence of *FBXO31* is colored based on conservation by Consurf server, and the variant is located in the conserved region (purple color)

To identify the disease‐causing variants, WES was performed on the proband (V‐2) and the family. Our results revealed a novel homozygous missense (ENST00000314349.4): c.766G>C, p. Glu256Gln) in the *TIMM50* gene in the proband (V‐2), located on chromosome 19. This variant in *TIMM50* (OMIM 617698) has changed the glutamate 256 residue to glutamine residue due to a single nucleotide substitution which is not presented in gnomAD genomes Iranome projects. According to 10 prediction tools from sDel_addAF, DANN, EIGEN, FATHMM‐MKL, LIST‐S2, M‐CAP, MutationAssessor (SCR_005762), MutationTaster (SCR_010777), PrimateAI, and SIFT (SCR_012813), it had a deleterious effect on the protein. The variant conformation was assessed by Sanger sequencing. Sanger data showed that other affected individuals were homozygous similar to the proband, while their mother and father were heterozygous carriers, and the unaffected individuals were a wild type or heterozygous (Figure 1C). These results showed that the diagnostic rate of a variant was exceeded in consanguineous families compared with non‐consanguineous families. Bioinformatics results predicted that the mutant amino acid could clash with other residues and increase the poor rotamers in the model, affecting the protein stability and conformation. (Figure 2B). Therefore, it could decrease the stability of protein according to DynaMut, mCSM, SDM, and DUET algorithms (Table 3).

**TABLE 3:** Predictions of E256Q and R511Q variants using several algorithms on stability protein

			E256Q	R511Q
No	Algorithm	Prediction	ΔΔG (kcal/mol)	
1	mCSM	Destabilizing	−1.292 kcal/mol	−0.994 kcal/mol
2	SDM	Destabilizing	−0.540 kcal/mol	−1.980 kcal/mol
3	DUET	Destabilizing	−1.197 kcal/mol	−1.295 kcal/mol
4	ENCoM	Destabilizing	−0.056 kcal/mol	−1.997 kcal/mol
5	DynaMut	Destabilizing	−0.439 kcal/mol	−1.410 kcal/mol

#### 3.1.3 Pedigree II

**FIGURE 3:**
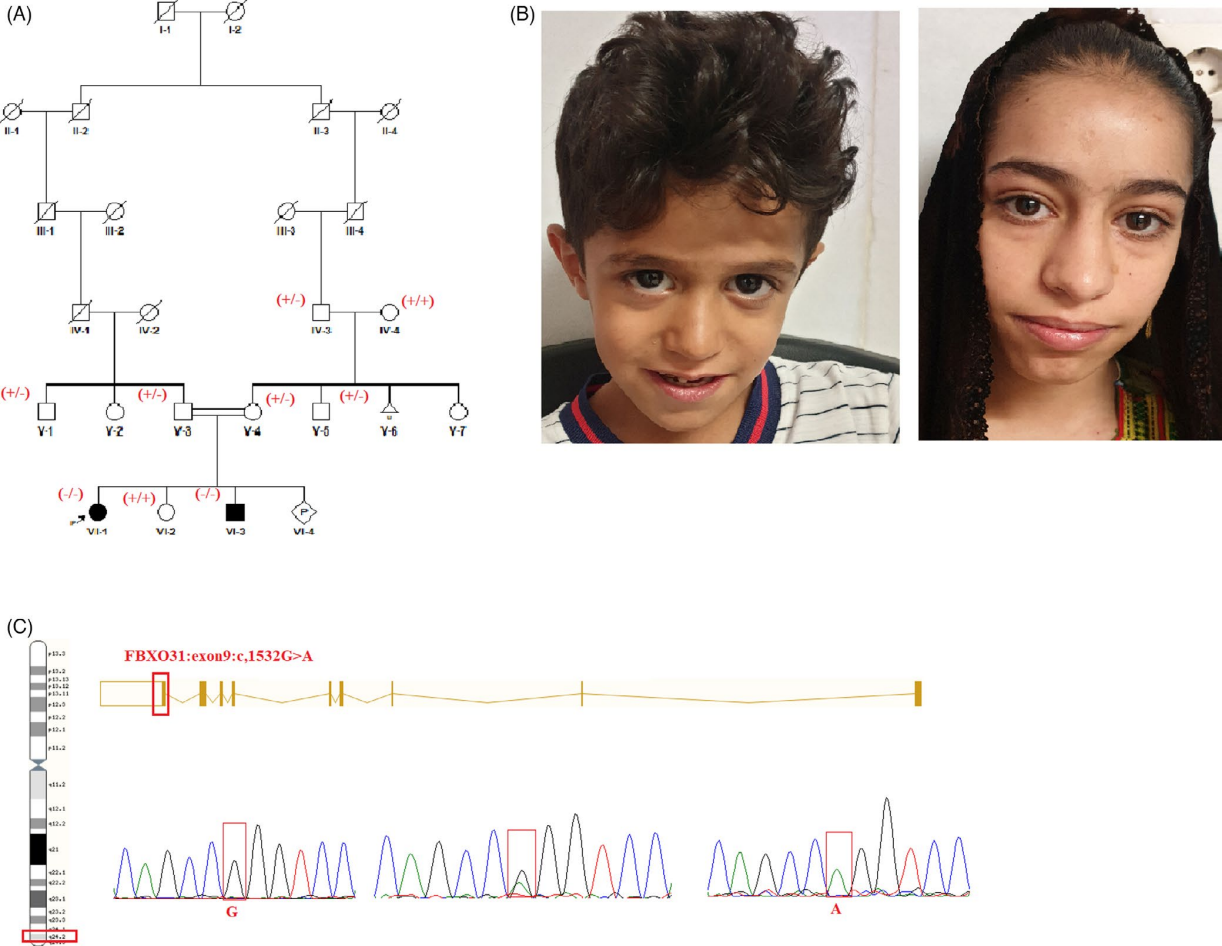
Pedigree of the family. (A) The family members with ID phenotype are shown in the family (in black) with segregation of the p.(Arg511Gln) variant in *FBXO31* gene. Squares represent males; circles stand for females; +/‐, heterozygote for p.(Arg511Gln) variant; ‐/‐ homozygous for p.(Arg511Gln) variant; +/+: wild type. Clinical features of the individual VI‐1 showing developmental delay, mild microcephaly, long face, prominent supraorbital ridges, fleshy nares, thick eyebrows, thick prominent lips, moderate intellectual disability, absent speech, and seizures. (B) Photographs of individual 1 and 2. (C) Sanger' results show mutant homozygous genotype (AA), heterozygous (AG), and wild‐type homozygous (GG) alleles in affected and healthy individuals, respectively, in the family

In this family (Figure 3A), 2 of 4 siblings were affected by the healthy consanguineous parents (VI‐1 and VI‐3 Figure 3B). The proband (VI‐1) in this family is a 14‐year‐old female who was assessed by both a genetic counselor and a physician in Ali‐Asghar hospital's genetic counseling center after presenting with ID symptoms. Her mother had a normal pregnancy and normal vaginal delivery at 45 weeks' gestation. There was a history of spontaneous abortion in her relatives. Clinical signs of the patient include developmental delay, mild microcephaly, long face, prominent supraorbital ridges, fleshy nares, thick eyebrows, thick prominent lips, moderate intellectual disability, absent speech, and seizures at the age of 3 months. Furthermore, the hearing was normal and there were no skeletal problems or coarse faces (Table 2). Her brother (VI‐3 aged 7 years) phenotype was like his older sister VI‐1. The laboratory assessments such as karyotype analysis and magnetic resonance imaging (MRI) were normal in the two affected individuals.

#### 3.1.4 Molecular analysis

Our bioinformatics results revealed a novel homozygous missense variant (c.1532G>A, p. Arg511Gln) in the *FBXO31* gene in the proband (VI‐1), located on chromosome 16 and composed of 9 exons. This variant in *FBXO31* (F‐Box Protein 31) (OMIM 615851) is not reported in the gnomAD and Iranome projects and is considered disease causing based on 11 prediction tools such as BayesDel_addAF, DANN, EIGEN, FATHMM‐MKL, LIST‐S2, M‐CAP, MutationAssessor, MutationTaster, and SIFT. To validate the variant in all affected and unaffected members of family, we used Sanger sequencing (described in the method section). The result showed the homozygous variant in her brother in the family, with unaffected family members being either heterozygous carriers, in particular the parents of the proband, or wild type (Figure 3C). Furthermore, our computational results predicted that the variant is located in the conserved region across different species and may alter the interaction between wild‐type and other residues (Figure 2C).

#### 3.1.5 Pedigree III

**FIGURE 4:**
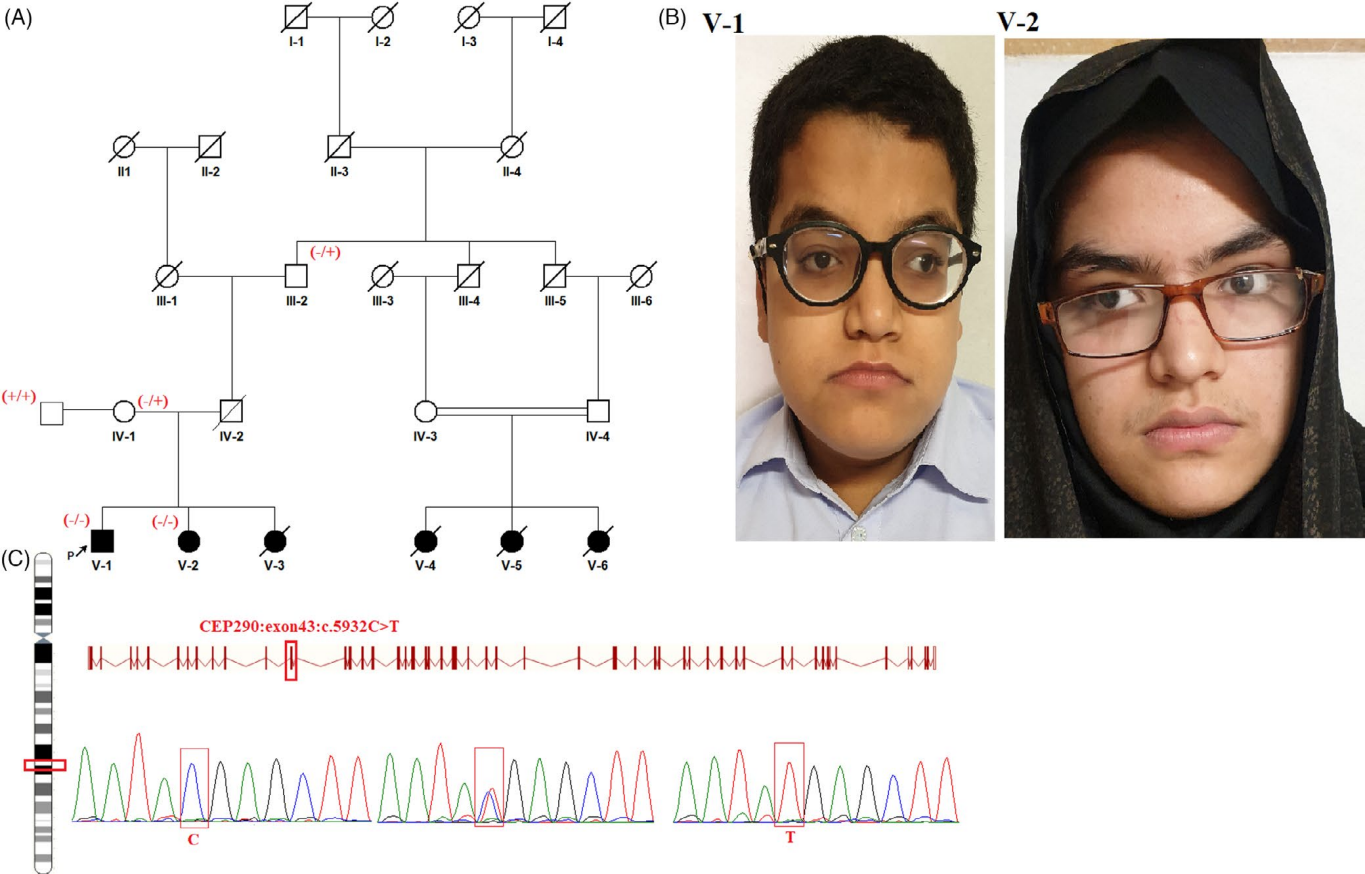
Pedigree III. (A) Consanguineous family with two affected individuals. (B) Photographes from these patients. (C) displays the segregation of c.5932C>T variants with the disease

In family III (Figure 4A), the proband, a 15 years old male (V‐1), showed a wide spectrum of clinical signs, including severe intellectual disability, seizures, developmental delay, lack of speech, obesity, visual impairment, and kidney disease (Table 2). His mother had a full‐term pregnancy and normal vaginal delivery with a birth weight of 3 kg and head circumference of 41 cm. He developed the first episodes of seizure at 3 months old, and afterward developed motor delay, and cognitive impairment. Brain MRI showed the cerebellar vermis aplasia at 7 years of age. Also, he developed congenital heart disease (arterial septal defect and ventricular septa defect) at the age of 1 year. Her sister (V‐2 aged 12 years) presented a similar phenotype to his older brother V‐1, but she had no vermis aplasia in brain MRI.

#### 3.1.6 Molecular analysis

The WES test for identifying any causative variants of the disease was executed for the probands. Our result revealed a missense variant (NM_025114.4: c.5932C>T; p. Arg1978Ter) in *CEP290* gene which was previously reported by Doherty et al (2015) (Table 4). The variant is a null variant in the gene, associated with several disorders such as Leber congenital amaurosis 10, Meckel syndrome 4, Senior‐Loken syndrome 6, Joubert syndrome 5, and Bardet‐Biedl syndrome 14. By helping in silico tools, it was determined that the variant had a damaging effect on the protein and disrupted the 3‐D structure of the protein (Figure 2A). Moreover, the variant segregated among all members of family (Figure 4C).

**TABLE 4:** Summary of the clinical features of the previously reported individuals with the mutations involved in FBXO31, TIMM50, and CEP290 genes

Authors	FBXO31						TIMM50					Cep290		
	Mir					Mir	Authors	Shahrour				Authors	Doherty	Elbadri Abdelgadir
Pt	Iv:3	Iv:4	Iv:8	Iv:9	Iv:10	‐	Pt	II−1	II−3	II−2	II−3	ID	+	+
ID	Moderate					Moderate	ID	Severe	Severe	Severe	Severe	Mutation	p. Arg1978Ter	c.5704G>T
Mutation	c.847‐852delinsA					Cys283nAs	Mutation	P. Thr252Met		P. Arg217Trp		Nephronophthisis	+	‐
Coarse facial	+	+	+	+	+	n.r	Spasm	+	+	‐	‐	Hypotonia	+	+
Brain imaging	‐	‐	n.r	n.r	n.r	n.r	Developmental delay	+	+	+	+	Cerebellar vermis hypoplasia	+	+
Broad nasal bridge	+	+	+	+	+	n.r	Hypotonia	+	+	‐	‐	Ocular apraxia	+	+
Fleshy nares	+	+	+	+	+	n.r	Seizures	+	+	+	+	Dysarthria	‐	+
Thick prominent lips	+	+	+	+	+	n.r	Failure to thrive	+	+	‐	+			
Round face shape	+	‐	‐	‐	‐	n.r	Speech	Poor	n.r	Poor	Poor			
Prominent supraorbital ridge	+	‐	‐	+	+	n.r	Visual impairment	+	+	‐	+			
Short Forehead	‐	+	‐	+	+	n.r	Brain atrophy	+	+	+	+			
Prominent eyebrows	‐	+	+	+	+	n.r	Elevated lactate	+	+	+	+			
Seizures	‐	‐	‐	‐	‐	n.r	Myoclonus	‐	‐	+	+			
In Silico						Pathogenic	Aggression	‐	‐	+	+			

Abbreviations: ‐, negative; +, positive; n.r, not reported.

## 4 DISCUSSION

Next‐generation sequencing was applied with some success to identify the rare causative variants of ID in consanguineous families, associated with the phenotype of the families.^19^ We identified two novel variants in *FBXO31* and *TIMM50* genes and one previously reported mutation in *CEP290* gene using WES in five consanguineous families diagnosed with autosomal recessive neurodevelopmental disorders with intellectual disability, confirmed through Sanger sequencing in their respective families.

A novel homozygous missense (c.766G>C, p. Glu256Gln) in *TIMM50* gene was found in six affected individuals with ID within three consanguineous families in pedigree II. This result showed that the diagnostic rate of a variant was exceeded in consanguineous families compared with non‐consanguineous families. *TIMM50* encodes a component of the translocase complex in the mitochondrial inner membrane. Some investigations showed that the protein was expressed intensively in the cerebral, cerebellar, and hippocampal cortices of rabbit brain.^20^ Knock out of *TIMM50* gene in fibroblast cells showed the abnormal release of cytochrome c, which is a crucial part of apoptosis.^21^ The missense variant, Glu256Gln, is predicted to be pathogenic via various in silico predictions. Defects in this gene are known to be associated with 3‐methylglutaconic aciduria (MGCA9) and variable complex V deficiency in these published cases.^22^ MGCA9 is a genetically heterogeneous disorder characterized by early‐onset seizures, developmental delay, intellectual disability, hypotonia, spasticity, increased serum lactate, and 3‐methylglutaconic aciduria, in which the clinical features are consistent with clinical characteristics of our cohort. The high level of serum lactate and 3‐methylglutaconic in laboratory tests suggested that there is a mitochondrial defect. The results of Sanger sequencing confirmed the segregation of mutation in a manner consistent with the clinical phenotypes of the affected individuals in the families, further proving causality. The clinical symptoms of patients related to intellectual disability molecularly confirmed diagnosis in the gene are listed in Table 4.

We found a novel homozygous missense variant (c.1532G>A) in *FBXO31* gene in pedigree I, which replaces the arginine residue with glutamine at position 511. *FBXO31* (F‐Box Protein 31) is a component of the SCF (SKP1‐cullin‐F‐box) complex which ligated the ubiquitin molecule to phosphorylated cyclin‐D1.^23^ Then, the complex acts as a cell cycle suppressor following DNA damage by an arrest at G1‐S checkpoint.^24^, ^25^ Some papers reported that the complex is essential for neuronal morphogenesis and axonal identification in the cerebellar cortex by the ubiquitination of the Par6c protein during brain development.^26^ Moreover, functional studies of *FBXO31* expression in mouse hippocampal neurons showed that the protein is located in axons and soma.^26^ Hence, mutations in the gene may disrupt the mechanism and result in neurodevelopment disorders like ID disease. The previously reported work proposed that a frameshift variant in the FBXO*31* gene causes mild‐to‐moderate intellectual disability and facial dysmorphisms such as broad nasal bridge, fleshy nares, thick eyebrows, and coarse faces in a family diagnosed with ID from Pakistan.^27^ The clinical features of our family are consistent with ID, but without the coarse faces phenotype, reported by Mir et al. (2014), which can be a result of different mutations in the gene. However, since there are not enough data, more studies will be needed to understand the pathophysiology mechanism of FBXO31 variants in ID disease.

In pedigree III, we observed a previously reported variant (c.5932C>T; p. Arg1978Ter) in *CEP290*, localized to the centrosome and cilia. As Doherty et al. (2015) reported, this mutation in the CEP290 gene was associated with Joubert syndrome, a genetic disorder characterized by cerebellar vermis hypoplasia, deep interpeduncular fossa, retinal dystrophy, and progressive renal failure.^28^ So far, our reassessment confirmed a consistent phenotype with Joubert Syndrome in the patient, segregating in the family. A brain MRI of his sister (with the same genotype) did not show the cerebellar vermis aplasia phenotype. Hence, it seems to be the main challenge in the diagnosis of patients.

In conclusion, we found two novel variants in the ID‐related genes *FBXO31* and *TIMM50* in the consanguineous families. The variants were confirmed in all affected and unaffected members of the families via Sanger sequencing, and their potential relevance to the disease was investigated using prediction tools. Therefore, the large and rare pedigrees can be helpful for us to identify ID diagnosis in the recruited families and expand our knowledge about potential mutation involved in the heterogeneous disease. To further confirm and asses the pathogenicity of the aforementioned variants, functional studies will be needed.

## CONFLICT OF INTEREST

The authors declare that they have no conflict of interest.

## AUTHOR CONTRIBUTION

Mohammadreza Dehghani, Seyed Mehdi Kalantar, and Mohsen Taheri conceptualized and designed the study. Zahra Metanat and Nasrin Ghasemi involved in data collection. Mohammad Yahya Vahidi Mehrjardi, Mahdiyeh Moudi, and Hossein Hozhabri analyzed and interpreted the results. Mahdiyeh Moudi and Hossein Hozhabri drafted the manuscript. The final version of the manuscript was reviewed, and the results were approved by all authors.

## CONSENT TO PARTICIPATE AND CONSENT TO PUBLISH

Informed consent was obtained from all family members before the study. Also, written informed consent was obtained from their parent for publishing their data and photographs.

## Data Availability

The data to support the findings in the study are available on request from the corresponding author.
